# Immunoglobulin G Fragment Crystallizable Glycosylation After Hematopoietic Stem Cell Transplantation Is Dissimilar to Donor Profiles

**DOI:** 10.3389/fimmu.2018.01238

**Published:** 2018-06-04

**Authors:** Noortje de Haan, Maarten J. D. van Tol, Gertjan J. Driessen, Manfred Wuhrer, Arjan C. Lankester

**Affiliations:** ^1^Center for Proteomics and Metabolomics, Leiden University Medical Center, Leiden, Netherlands; ^2^Department of Pediatrics, Section Immunology, Hematology and Stem Cell Transplantation, Leiden University Medical Center, Leiden, Netherlands; ^3^Department of Pediatrics, Juliana Children’s Hospital, Haga Teaching Hospital, The Hague, Netherlands; ^4^Department of Pediatrics, Erasmus Medical Center, Sophia Children’s Hospital, Rotterdam, Netherlands

**Keywords:** immunoglobulin G, fragment crystallizable glycosylation, *N*-glycan, hematopoietic stem cell transplantation, immune reconstitution

## Abstract

Immunoglobulin G (IgG) fragment crystallizable (Fc) *N*-glycosylation has a large influence on the affinity of the antibody for binding to Fcγ-receptors (FcγRs) and C1q protein, thereby influencing immune effector functions. IgG Fc glycosylation is known to be partly regulated by genetics and partly by stimuli in the microenvironment of the B cell. Following allogeneic hematopoietic stem cell transplantation (HSCT), and in the presence of (almost) complete donor chimerism, IgG is expected to be produced by, and glycosylated in, B cells of donor origin. We investigated to what extent IgG glycosylation in patients after transplantation is determined by factors of the donor (genetics) or the recipient (environment). Using an IgG subclass-specific liquid chromatography–mass spectrometry method, we analyzed the plasma/serum IgG Fc glycosylation profiles of 34 pediatric patients pre-HSCT and at 6 and 12 months post-HSCT and compared these to the profiles of their donors and age-matched healthy controls. Patients treated for hematological malignancies as well as for non-malignant hematological diseases showed after transplantation a lower Fc galactosylation than their donors. Especially for the patients treated for leukemia, the post-HSCT Fc glycosylation profiles were more similar to the pre-HSCT recipient profiles than to profiles of the donors. Pre-HSCT, the leukemia patient group showed as distinctive feature a decrease in sialylation and in hybrid-type glycans as compared to healthy controls, which both normalized after transplantation. Our data suggest that IgG Fc glycosylation in children after HSCT does not directly mimic the donor profile, but is rather determined by persisting environmental factors of the host.

## Introduction

The *N*-glycan attached to the conserved glycosylation site of the fragment crystallizable (Fc) region of immunoglobulin G (IgG) has a large influence on the structure and function of the antibody (1, 2). The presence or absence of specific monosaccharides have proven to be crucial both in modulating the affinity of IgG binding to Fcγ-receptors (FcγRs) and in the activation of the complement system. For example, the absence of a core fucose on the Fc glycan results in a 10- to 20-fold increase in binding affinity to the FcγRIIIa and FcγRIIIb and a corresponding increase in antibody-dependent cellular cytotoxicity (2, 3). For IgG Fc binding to complement factor C1q, galactosylation plays a primary role and is associated positively with binding affinity and downstream complement-dependent cytotoxicity (CDC) (3, 4).

Besides the functional implications of altered IgG Fc glycosylation described above, the IgG Fc glycosylation profile was found to be associated with altered physiological states and disease activity. For example, galactosylation decreases with age in the adult population (5). In children, on the other hand, Fc galactosylation remains relatively constant with age, while bisection increases and fucosylation and sialylation decrease (6). Furthermore, in adults, total IgG-Fc galactosylation is decreased in rheumatoid arthritis and active tuberculosis infections (7, 8), but also in different types of cancer, like ovarian cancer and colorectal cancer (9, 10). Antigen-specific IgG antibodies may display discriminative glycosylation patterns. For example, gp120-specific antibodies in HIV-infected patients show a decrease in fucosylation as compared to the total pool of IgG in these patients (11).

Immunoglobulin G Fc *N*-glycans are co-translationally attached to the protein in the endoplasmic reticulum (ER) of B lymphocytes and enzymatically shaped into their final structure in the ER and Golgi apparatus. This process is dependent on the time the protein spends in the Golgi apparatus, the abundance of monosaccharide donors and presence of specific glycosyltransferases and glycosidases, adding and removing particular monosaccharides to and from the glycoconjugates, respectively (12). All these factors are known to be partly regulated by the genetics of the B cell (13, 14), and on the other hand, by external stimuli in the microenvironment of the cells, like hormones and cytokines (15, 16). For example, in *in vitro* experiments, all-*trans* retinoic acid and human interleukin (IL)-21 cause a decrease and increase in both IgG1 Fc galactosylation and sialylation, respectively (16). However, the exact mechanisms leading to specific IgG Fc glycosylation profiles are not fully understood yet. Unraveling the up-stream factors influencing IgG glycosylation will improve the understanding of changes hereof that are associated with immune-related diseases.

Allogeneic hematopoietic stem cell transplantation (HSCT) is a curative treatment for patients with malignant as well as non-malignant immune-hematological diseases. In addition to the cure of the primary disease, proper immune reconstitution is an important goal of any HSCT procedure. Previous studies in non-transplant patients have demonstrated that IgG Fc glycosylation patterns are strongly influenced by both B cell intrinsic and (host) environmental factors (13, 15, 16). HSCT provides a unique setting to study the impact of both determinants on IgG Fc glycosylation in transplanted patients. With this aim, we studied a group of pediatric patients that were successfully treated for their initial disease, and in whom (close to) complete donor chimerism was documented at 6 and 12 months post-transplant. Furthermore, the patient group was homogeneous in terms of reaching steady state within this timeframe, i.e., the presence of an uncomplicated clinical condition without requirement of any immunomodulatory medication.

The IgG Fc glycosylation profiles of transplant recipients were analyzed before and after HSCT and compared to the profiles of the donors as well as to those of age-matched healthy controls.

## Materials and Methods

### HSCT Patients

In the period 2010–2014, 211 allogeneic HSCT procedures were performed in children at the pediatric transplantation unit of the LUMC. Criteria for exclusion of patients to enroll in the current study were: death within 1 year after HSCT, an eventful course in the first year after HSCT such as relapse of the original disease, acute graft-vs.-host disease (GvHD) grade >1 or extensive chronic GvHD, dependency of IgG supplementation within a period of 2 months before taking a serum or plasma sample at 6 and 12 months after HSCT and dependency of immunosuppressive drugs (i.e., cyclosporine A) at 7 months after HSCT (median 3.8 months). In addition, patients treated for thalassemia and with a persistent mixed chimerism in peripheral blood mononuclear cells (PBMC) defined as <85% donor origin at 1-year post-HSCT were excluded. Finally, to be included in the study, at least three of the four following serum or plasma samples of a donor-recipient pair should be available: from the graft donor, from the patient before HSCT (and start of the conditioning), and at 6 and 12 months after HSCT.

The final study cohort consisted of 34 pediatric HSCT recipients. In Table 1, the characteristics of these patients and their donors are summarized. All transplantation procedures were performed according to national protocols and in line with the recommendations of the European group for Blood and Marrow Transplantation.

**Table 1:** Summary of the cohort.

Patients			
Group	Malignant HD^a^	Non-malignant HD^a^	Total
Number (*N*) of patients	16	18	34
Age at HSCT^b^ (median, range)	9.4 (0.8–17.8)	5.7 (0.7–14.9)	7.5 (0.7–17.8)
Sex recipient (% female)	31.3	16.7	23.5
Donor type:
IRD (BM/CB)	4 (4/0)	11 (10/1)	15 (14/1)
MUD (BM/CB)	10 (9/1)	6 (3/3)	16 (12/4)
ORD (PBSC)	2 (2)	1 (1)	3 (3)
Donor age^b^ (median, range)	26.6 (8.9–49.0)	17.1 (2.8–47.5)	25.6 (2.8–49.0)
Sex donor (% female)	12.5	35.7	24.1
Graft: undepl. (*N*)/CD34-enriched (*N*)	14/2	17/1	31/3
% chimerism^c^ (median, range)	100 (98–100)	100 (87–100)	100 (87–100)
Acute GvHD (grade 1, *N*)	2	1	3
Chronic GvHD (limited, *N*)	1	0	1
1st sample (*N*)	16	16	32
1st sample^d^ (median, range)	−13 (−21 to −6)	−14 (−28 to −6)	−13 (−28 to −6)
2nd sample (*N*)	16	18	34
2nd sample^d^ (median, range)	176 (162–199)	183 (158–205)	182 (158–205)
3rd sample (*N*)	16	18	34
3rd sample^d^ (median, range)	375 (348–499)	365 (313–431)	372 (313–499)
Donor sample (*N*)	15	14	29
Donor sample^d^ (median, range)	−1 (−159 to 0)	−24 (−43 to 0)	−14 (−159 to 0)

**Controls**			

**Group**	**Age matched to malignant HD**	**Age matched to non-malignant HD**	**Total**

Controls (*N*)	16	18	81
Age (median, range)	9.3 (0.8–16.8)	6.0 (0.6–13.8)	6.2 (0.5–16.8)
Sex (% female)	68.8	27.8	49.4

*^a^HD, hematological disease; malignant HD: acute lymphoblastic leukemia: N = 10; myelodysplastic syndrome/acute myeloblastic leukemia: N = 2; acute myeloblastic leukemia: N = 4. Non-malignant HD: thalassemia: N = 4, Fanconi anemia: N = 3, sickle cell disease: N = 2, severe aplastic anemia: N = 1, progressive bone marrow failure: N = 1, neutropenia congenita: N = 1, Glanzmann thrombasthenia: N = 1, hemophagocytic lymphohistiocytosis: N = 4, X-linked lymphoproliferative disease: N = 1*.

*^b^Age at hematological stem cell transplantation and graft donation (excluding cord blood donors), respectively, in years*.

*^c^% donor chimerism in peripheral blood mononuclear cells (PBMC) at 1-year post-HSCT*.

*^d^Day of serum/plasma sampling relative to the day of HSCT*.

*IRD, identical related donor; MUD, matched unrelated donor; ORD, other than HLA-genotypically identical related donor; BM, bone marrow; CB, cord blood; PBSC, peripheral blood stem cells; GvHD, graft-vs.-host disease*.

### Sampling and Study Approval

Serum or plasma samples of HSCT recipients were collected between 6 and 28 days (median 13 days) prior to HSCT, i.e., before start of conditioning, and 6 months (range 158–205 days) and 12 months (range 313–499 days) after HSCT. From 29 of the stem cell donors (excluding umbilical cord donors) a plasma or serum sample was collected between 0 and 159 days (median 14 days) before donation of the graft (Table 1). All clinical samples were collected with approval of the Medical Ethics Committee of the LUMC, Leiden (project P01.028) and after receiving written informed consent from the participants. In addition, samples were collected from 81 healthy controls in the same age range as the patients. The healthy control samples were collected with approval of the Medical Ethics Committee of the Erasmus MC, Rotterdam (MEC-2005-137) and after receiving written informed consent from the participants (6, 17).

### IgG Glycopeptide Analysis and Data Processing

All 135 clinical samples (patients and donors) were randomized in 96-well plates, together with 12 plasma standards (VisuCon pooled plasma; Affinity Biologicals Inc., Ancaster, ON, Canada) and 5 PBS blanks. Healthy control samples were randomized on separate plates, including 29 plasma standards and 8 PBS blanks. IgG was isolated using Protein G affinity beads (GE Healthcare, Uppsala, Sweden) and digested by trypsin as described before (18), see Supplementary Materials and Methods in Supplementary Material. The IgG digest was separated and analyzed by nano-liquid chromatography (LC) coupled by electrospray ionization to a Maxis Impact HD quadrupole time-of-flight mass spectrometer (q-TOF-MS; Bruker Daltonics, Bremen, Germany) as described before (18), see Supplementary Materials and Methods in Supplementary Material. Prior to statistical analysis, raw LC–MS data were extracted and curated using the in-house developed software LacyTools v0.0.7.2 as described previously (18, 19); for all cohort-specific extraction parameters see Supplementary Materials and Methods in Supplementary Material.

### Statistics

**Figure 1:**
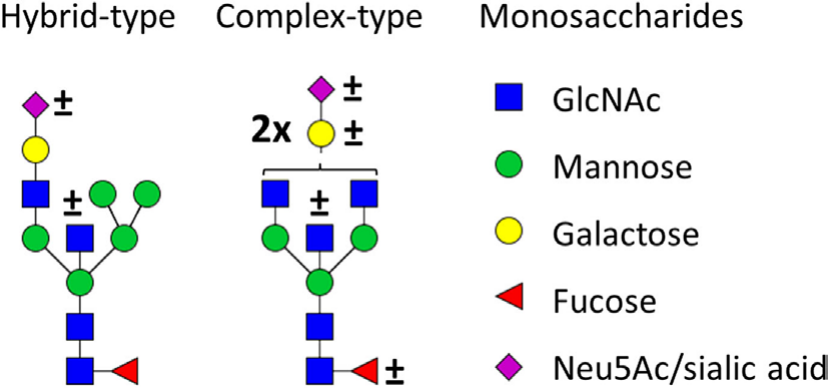
Glycan structures found on immunoglobulin G (IgG)-fragment crystallizable (Fc). Both hybrid-type and diantennary complex-type glycan structures were found on the IgG Fc glycopeptides in our study. Hybrid-type glycans varied by the presence of a sialic acid and/or a bisecting *N*-acetylglucosamine (GlcNAc). Diantennary complex-type glycans were found with zero to two galactoses, zero to two sialic acids, and the presence or absence of a bisecting GlcNAc and/or a core fucose. Monosaccharide linkages were based on literature (6, 13, 20, 21). Green circle: mannose, yellow circle: galactose, blue square: GlcNAc, red triangle: fucose, pink diamond: *N*-acetylneuraminic acid (Neu5Ac/sialic acid).

**Figure 2:**
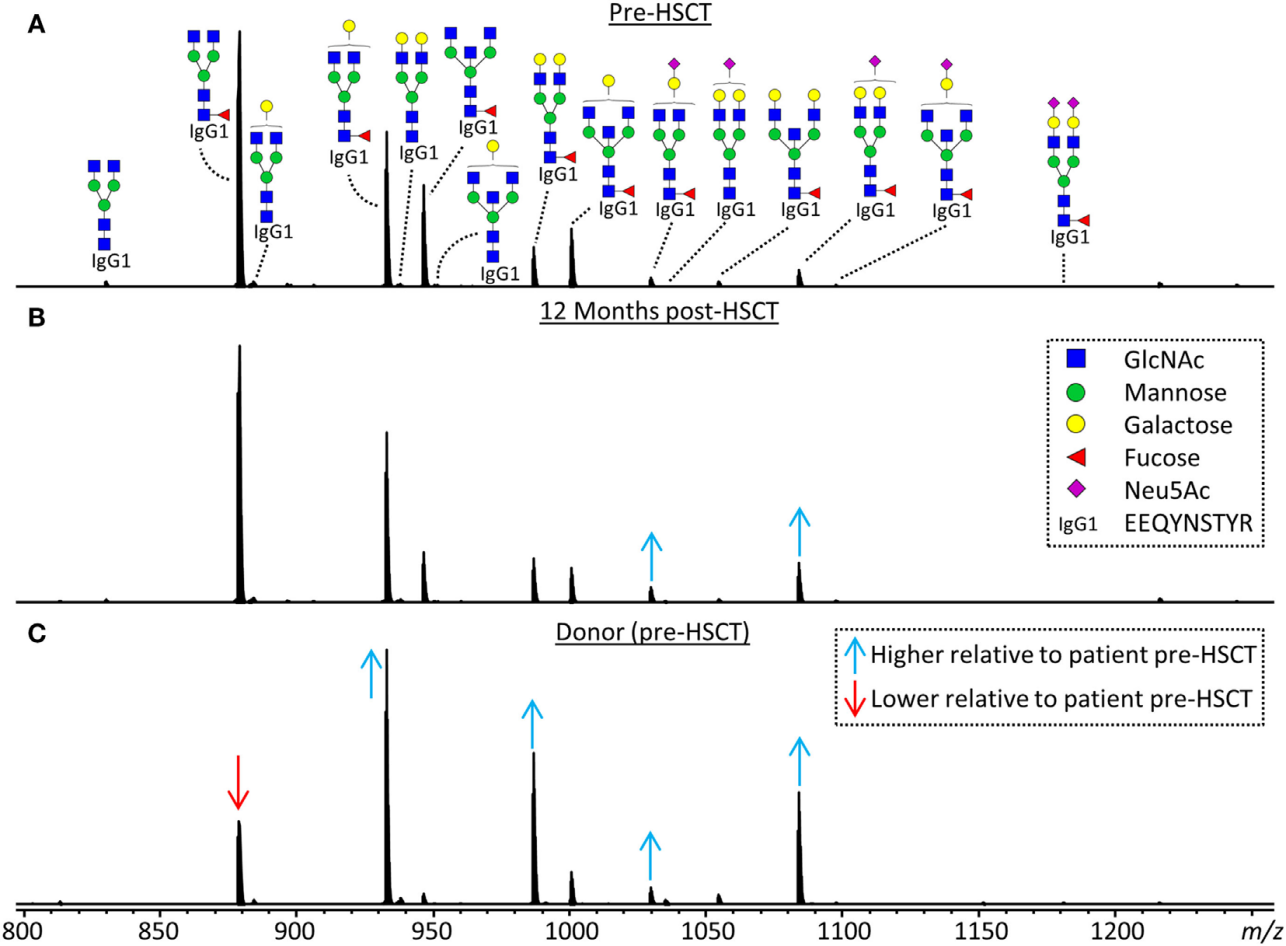
Representative mass spectra of IgG1 glycopeptides. Annotated are the 15 most abundant IgG1 glycoforms detected for a 7.5 years old, male, acute myeloid leukemia patient **(A)** before hematopoietic stem cell transplantation (HSCT), **(B)** 12 months after HSCT and **(C)** his 24 years old, healthy, male donor. Triply charged glycopeptides are shown, the proposed glycan structures are based on literature (6, 13, 20, 21). Green circle: mannose, yellow circle: galactose, blue square: *N*-acetylglucosamine (GlcNAc), red triangle: fucose, pink diamond: *N*-acetylneuraminic acid (Neu5Ac). Differences in relative abundances of glycoforms as compared to the patient pre-HSCT are indicated by red (lower than in pre-HSCT patient) and blue (higher than in pre-HSCT patient) arrows.

The absolute intensities of the extracted glycoforms (Figures 1 and 2; Table S1 in Supplementary Material) were total-area normalized per IgG subclass and levels of galactosylation, sialylation, fucosylation, as well as other derived traits were calculated (Table 2; Table S2 in Supplementary Material). To assess data quality throughout the cohort measurements, the plasma standards were analyzed, revealing highly repeatable glycosylation profiles for all subclasses, with median relative SD of all extracted glycoforms of IgG1, IgG2/3, and IgG4 being 3.4, 2.7, and 2.1%, respectively (Figure S1 in Supplementary Material). For three patients, both plasma and serum samples, obtained at corresponding time points, were available. Comparison of these samples showed similar IgG Fc glycosylation profiles for both materials (Figure S2 in Supplementary Material). The difference between measurements in plasma and serum from the same patient was lower than the variation between patients, enabling the use of both materials in this study.

**Table 2:** Subclass-specific-derived traits.

Derived trait^a^	Depiction^b^	Description^c^
Hybrids	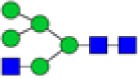	Fraction of hybrid-type glycans
Bisection	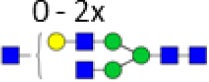	Fraction of glycans with a bisecting *N*-acetylhexosamine
Fucosylation	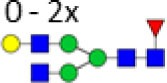	Fraction of glycans with a core fucose
Galactosylation	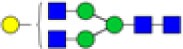	Galactosylation per antenna of diantennary glycans
Sialylation	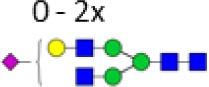	Sialylation per antenna of diantennary glycans
Sialylation per galactose	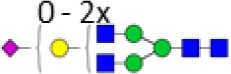	Sialylation per galactose of diantennary glycans

*^a^The individual glycoforms were grouped based on their glycosylation features as described before for immunoglobulin G glycopeptides in human (6)*.

*^b^Green circle: mannose, yellow circle: galactose, blue square: N-acetylglucosamine, red triangle: fucose, pink diamond: N-acetylneuraminic acid. The depictions of the derived traits show the minimally required composition to contribute to a trait*.

*^c^For detailed calculations of the traits, see Table S2 in Supplementary Material*.

Further statistical analysis and data visualization were performed using R 3.1.2 (R Foundation for Statistical Computing, Vienna, Austria) and RStudio 0.98.1091 (RStudio, Inc., Boston, MA, USA). Patients pre- and 12 months post-HSCT, as well as patients and donors, were compared with two-sided Wilcoxon signed-rank tests. Healthy controls and patients were compared with two-sided Mann–Whitney *U* tests. The samples of the patients taken 6 months post-HSCT were left out of the statistical analysis to reduce the data density, but were shown in some figures to illustrate the dynamics of IgG Fc glycosylation profiles after HSCT. Statistical tests were performed for the whole dataset, as well as after stratification on diagnosis: non-malignant hematological disease and malignant hematological disease (Table 1). For the tests after stratification for diagnosis, subgroups of one-to-one age-matched healthy controls were used. A significance threshold (α) = 0.015 was used throughout the study after correcting for multiple testing using the Benjamini–Hochberg approach with an FDR of 5%.

## Results

The IgG Fc glycosylation profiles of 34 pediatric HSCT patients were followed over time, starting with a sample prior to the HSCT and followed by two longitudinal samples, 6 and 12 months post-HSCT. In addition, the IgG Fc glycosylation of the donors prior to the donation of the grafts and of age-matched healthy controls was assessed (Table 1). IgG Fc glycopeptides were analyzed by LC–MS, which enabled the detection of 22 glycoforms on IgG1, 16 on IgG2/3, and 11 on IgG4 (Figures 1 and 2; Table S1 in Supplementary Material). Furthermore, derived glycosylation traits, such as levels of galactosylation, sialylation, fucosylation, and bisection were calculated (Table 2; Table S2 in Supplementary Material).

### IgG Fc Glycosylation Differences Between Patients and Healthy Controls

**Figure 3:**
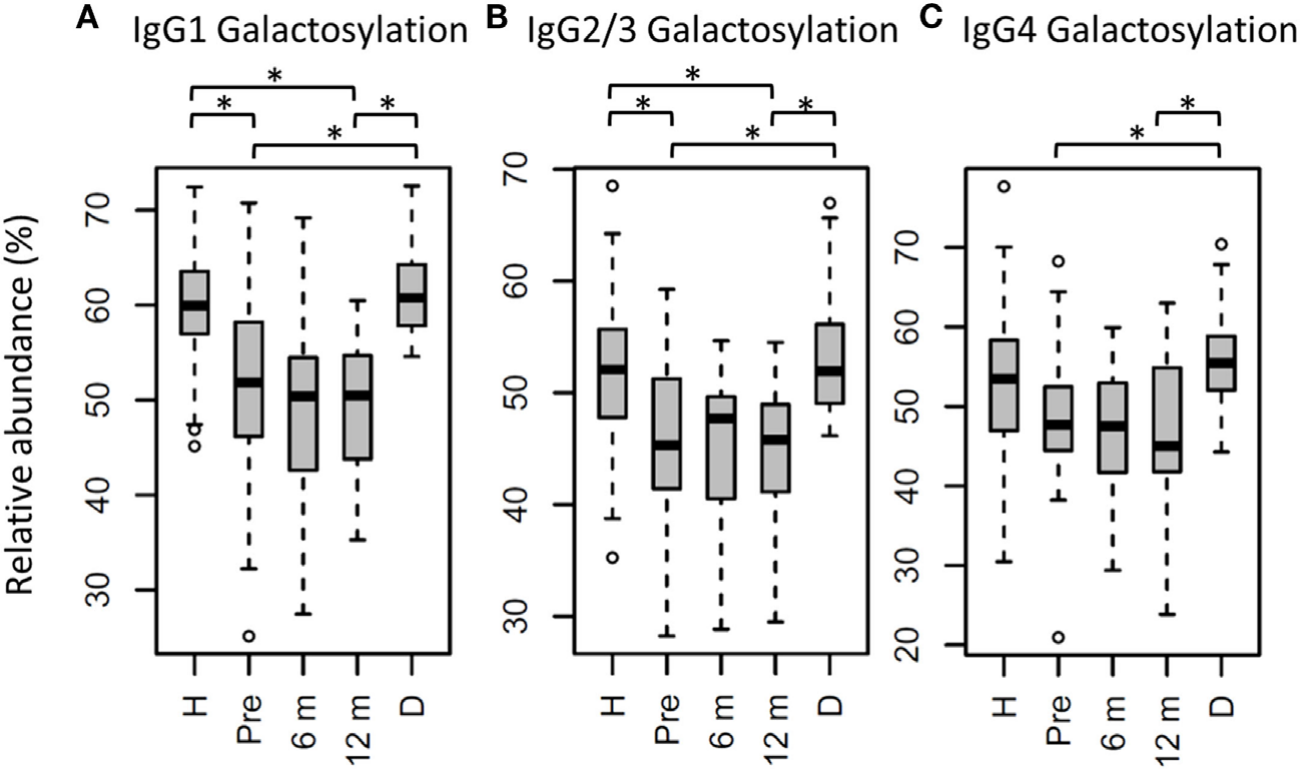
Immunoglobulin G (IgG) fragment crystallizable (Fc) galactosylation in all patients, compared to their donors and age-matched healthy controls. IgG Fc galactosylation on **(A)** IgG1, **(B)** IgG2/3, and **(C)** IgG4 is different between patients before hematopoietic stem cell transplantation (HSCT) (Pre) and their donors (D). No changes were observed between patients pre- and 12 months post-HSCT (12 m). In general, patients before and after HSCT have lower galactosylation than age-matched healthy controls (H), although this was not statistically significant for IgG4 galactosylation. 6 m: patients 6 months after HSCT. Patients and donors were compared by Wilcoxon signed-rank tests, healthy controls and patients with Mann–Whitney *U* tests. *p*-Values <0.015 were considered statistically significant after 5% FDR correction (as indicated by the asterisks above the plots). For IgG1 galactosylation, *n* (H, Pre, 6 m, 12 m, D) = 81, 32, 34, 34, 29, for IgG2/3 galactosylation, *n* (H, Pre, 6 m, 12 m, D) = 69, 32, 32, 33, 29, for IgG4 galactosylation, and *n* (H, Pre, 6 m, 12 m, D) = 59, 27, 15, 17, 28, respectively.

For the total patient group pre-HSCT, IgG Fc bisection was higher than for healthy controls (e.g., IgG1: 14.8 vs. 10.1%, respectively, *p* = 2.5*10^−7^; Table S3 and Figure S3 in Supplementary Material), while IgG1 and IgG2/3 galactosylation and sialylation were lower (e.g., IgG1 galactosylation: 51.9 vs. 59.9%, *p* = 3.2*10^−6^; Figure 3; Table S3 and Figure S3 in Supplementary Material). Furthermore, specifically on IgG1, fucosylation and hybrid-type glycans were lower in patients than in controls (fucosylation: 95.6 vs. 97.5%, *p* = 1.1*10^−5^, and hybrid-types: 0.3 vs. 0.4%, *p* = 6.6*10^−3^; Table S3 and Figure S3 in Supplementary Material).

**Figure 4:**
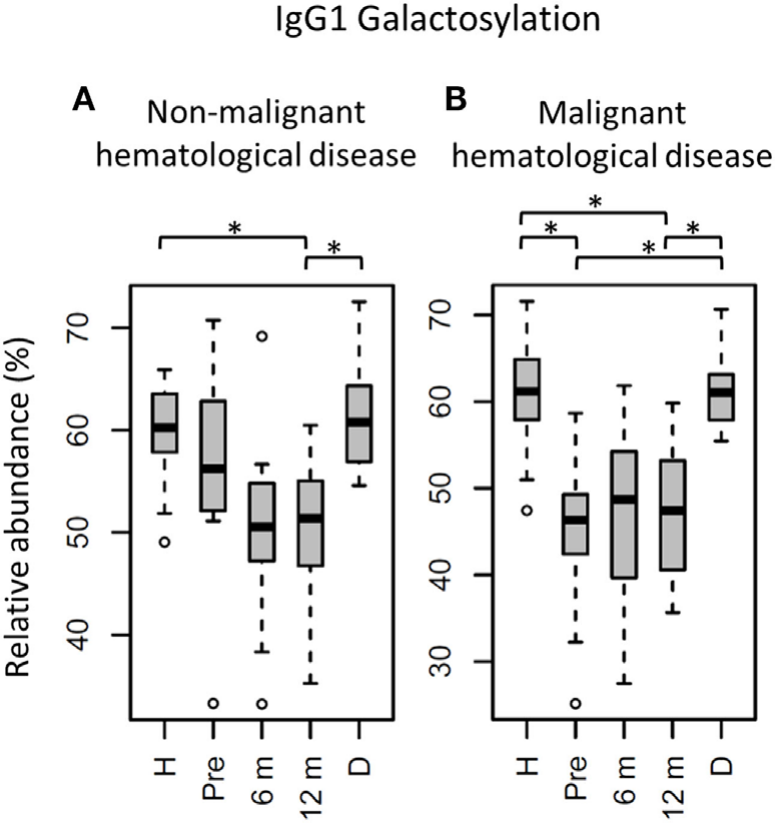
IgG1 fragment crystallizable (Fc) galactosylation in all patients stratified on diagnose, compared to their donors and age-matched healthy controls. IgG1 Fc galactosylation in children **(A)** with non-malignant hematological diseases and **(B)** with malignant hematological diseases. H: age-matched healthy controls, Pre: patients prior to hematopoietic stem cell transplantation (HSCT), 6 m: patients 6 months after HSCT, 12 m: patients 12 months after HSCT, D: donors prior to HSCT. Patients and donors were compared by Wilcoxon signed-rank tests, healthy controls and patients with Mann–Whitney *U* tests. *p*-Values <0.015 were considered statistically significant after 5% FDR correction (as indicated by the asterisks above the plots). For non-malignant hematological diseases, *n* (H, Pre, 6 m, 12 m, D) = 18, 16, 18, 18, 14, for malignant hematological diseases, *n* (H, Pre, 6 m, 12 m, D) = 16, 16, 16, 16, 15, respectively.

**Figure 5:**
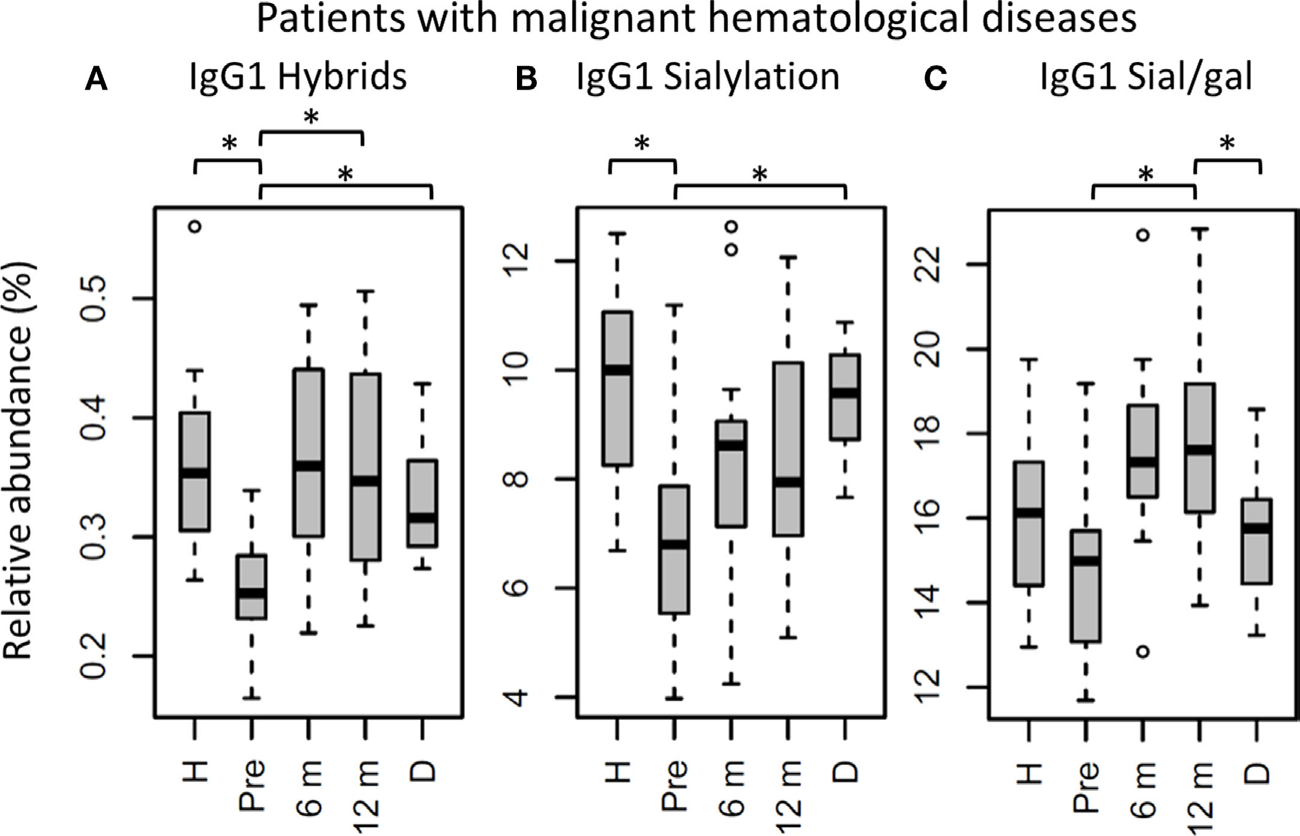
IgG1 fragment crystallizable (Fc) glycosylation features in patients with malignant hematological diseases, compared to their donors and age-matched healthy controls. IgG1 **(A)** hybrid-type glycans and **(B)** sialylation differ between healthy controls (H) and patients before hematopoietic stem cell transplantation (HSCT) (Pre). In addition, IgG1 **(A)** hybrid-type glycans and **(B)** sialylation are different between patients and donors (D), and IgG1 **(A)** hybrid-type glycans and **(C)** sialylation per galactose change within the patients after HSCT. 6 m: patients 6 months after HSCT, 12 m: patients 12 months after HSCT. Patients and donors were compared by Wilcoxon signed-rank tests, healthy controls and patients with Mann–Whitney *U* tests. *p-*Values <0.015 were considered statistically significant after 5% FDR correction (as indicated by the asterisks above the plots). For all IgG1 glycosylation features in patients treated for malignant hematological diseases, *n* (H, Pre, 6 m, 12 m, D) = 16, 16, 16, 16, 15, respectively.

The patients were grouped according to their diagnosis, resulting in a group with malignant and with non-malignant hematological diseases (Table 1). These groups differ in disease course and treatment before HSCT, and were hence compared separately to healthy controls with respect to IgG Fc glycosylation (Tables S4 and S5 in Supplementary Material). The pre-HSCT malignant hematological disease group differed in IgG Fc glycosylation from age-matched healthy controls similar to the overall pre-HSCT patient group (Figures 4 and 5; Table S4 in Supplementary Material). In contrast, the pre-HSCT non-malignant hematological disease group only showed a decrease in IgG1 fucosylation as compared to age-matched healthy controls (Table S5 and Figure S5 in Supplementary Material).

### The Impact of HSCT on IgG Fc Glycosylation

In the whole dataset, pre-HSCT IgG Fc glycosylation differed from the corresponding donors (Table S3 and Figure S3 in Supplementary Material). For all subclasses, IgG galactosylation was higher in donors than in recipients pre-HSCT (IgG1: 60.8 vs. 51.9%, *p* = 5.5*10^−6^; Figure 3). In addition, IgG1 fucosylation was lower in donors (94.1%) than in recipients pre-HSCT (95.6%, *p* = 1.0*10^−3^), while IgG1 sialylation was higher in donors (10.0%) than in recipients (8.5%, *p* = 1.5*10^−3^; Table S3 and Figure S3 in Supplementary Material). Both galactosylation and IgG1 sialylation were still lower in patients 12 months post-HSCT as compared to the donors, and no difference in galactosylation and sialylation was observed between the patients pre-HSCT and 12 months post-HSCT (Figure 3; Table S3 and Figure S3 in Supplementary Material). The differences in IgG Fc glycosylation between pre-HSCT patients and healthy controls, as described above, were still observed 12 months post-HSCT, except for IgG1 hybrid-type glycans and IgG2/3 sialylation which both normalized (Table S3 in Supplementary Material).

The effect of HSCT on IgG Fc glycosylation was studied additionally for the two diagnosis groups separately (Tables S4 and S5 and Figures S4 and S5 in Supplementary Material). The observations made on IgG Fc galactosylation in the total dataset, were found back in the patients treated for malignant hematological diseases, but only partly in the other subgroup (Figure 4). For the patients treated for hematological malignancies, galactosylation was lower for the recipients pre-HSCT (46.3%) than for the donors (IgG1: 61.0%, *p* = 1.2*10^−4^). The reduced galactosylation in recipients was still observed 12 months post-HSCT (47.4%, *p* = 6.1*10^−5^), while no difference was observed between patients before and after HSCT (Figure 4; Table S4 in Supplementary Material). Another glycosylation trait that was found to be overall low, but significantly lower only in pre-HSCT patients with a hematological malignancy as compared to their donors, was the relative presence of the IgG1 hybrid-type glycans (recipient: 0.25% and donor: 0.32%, *p* = 1.2*10^−4^). In contrast to galactosylation, the abundance of hybrid-type glycans normalized after HSCT (0.35%; Figure 5A; Table S4 in Supplementary Material). In addition, IgG1 sialylation was only lower in the patients with a hematological malignancy pre-HSCT (6.8%) as compared to their donors (9.6%, *p* = 3.1*10^−4^), and showed an increasing trend after HSCT (Figure 5B). This increase in IgG1 sialylation was more pronounced when assessed per galactose (pre-HSCT: 15.0%, 12 months post-HSCT: 17.6%, *p* = 1.5*10^−3^) and sialylation per galactose was higher after HSCT (17.6%) than in the corresponding donors (15.8%, *p* = 1.2*10^−4^; Figure 5C).

The group treated for non-malignant diseases showed no differences between donors and recipients pre-HSCT (Figure 4; Table S5 in Supplementary Material). However, a decrease in fucosylation was observed after HSCT (IgG1-pre: 96.3%, post: 95.5%, *p* = 1.2*10^−2^; Table S5 and Figure S5 in Supplementary Material). In addition, IgG1 fucosylation and galactosylation were lower post-HSCT than for age-matched healthy controls (fucosylation: 95.5 vs. 97.9%, *p* = 6.6*10^−4^, and galactosylation: 51.4 vs. 60.2%, *p* = 6.0*10^−6^; Figure S5 in Supplementary Material), while bisection was higher for the post-HSCT patients than compared to the controls (13.5 and 10.1%, *p* = 1.1*10^−4^, respectively). Of note, the post-HSCT levels of galactosylation (IgG1: 51.4%) were also lower than those found in the donors (60.8%, *p* = 9.8*10^−4^; Figure S5 and Table S5 in Supplementary Material).

## Discussion

### Recipient Galactosylation Is Different From Donor Galactosylation

Here, we studied IgG Fc glycosylation in pediatric patients before and after HSCT as compared to their donors. Looking at the whole dataset, the most prominent IgG Fc glycosylation differences between donors and recipients pre-HSCT were observed in the galactosylation, which was lower in the patients as compared to the donors independently of the IgG subclass. This cannot be explained by the age difference between the groups, as galactosylation does hardly differ between children and young adults (6, 22). Furthermore, a significant difference remained present for the galactosylation of IgG1 and IgG2/3 when comparing recipients pre-HSCT with healthy age-matched controls. Therefore, a low galactosylation state most likely reflects glycosylation changes caused by the disease and/or the treatment thereof. This is supported by the observation that, when the patients were stratified based on diagnosis, only the children treated for hematological malignancies showed a lower galactosylation as compared to both their donors and the healthy age-matched controls. IgG Fc galactosylation was reported before to be lowered in patients with solid tumors (9, 10).

Twelve months after HSCT, the patients treated for hematological malignancies still showed a lower IgG Fc galactosylation as compared to their donors, which indicates that the recipients produce IgG with a Fc glycosylation pattern that partly mimics the profile of the recipient pre-HSCT and not the donor profile. Of note, the patient-subgroup treated for non-malignant hematological diseases did also show a low galactosylation 12 months post-HSCT, while their levels did not differ from the donor levels at the pre-HSCT timepoint. The low level of galactosylation at this stage after HSCT, when all study patients were in a stable and uncomplicated clinical condition, was comparable between the two subgroups. A likely explanation for this aberrant IgG Fc galactosylation is, at least partly, the regulation of this Fc glycosylation trait by host microenvironmental factors like cytokines or hormones which persists for a prolonged period after HSCT. In this respect, it would be interesting to study the expression of glycan-modifying enzymes, like glycosyltransferases and glycosidases, and influences thereof, both before and after HSCT. While the used chemotherapeutic medication (for example, clofarabine) has been reported to perform their function through epigenetic mechanisms, it seems unlikely that this influences the IgG production by the donor B cells as these drugs are administered to the patients before the HSCT and are cleared from the circulation rapidly.

### Hybrid-Type Glycans and Sialylation Increase After HSCT

While aberrant galactosylation in patients treated for hematological malignancies did not change after HSCT, IgG1 hybrid-type glycans and sialylation per galactose increased to a normal level after HSCT in these patients. Although for all samples in the cohort the total relative abundance of the hybrid-type glycans was below 1% of the total glycan profile, still a clear increase was observed in the proportion of hybrid-type glycans after HSCT for hematological malignancies. Hybrid-type structures are intermediate in the glycosylation biosynthesis, emerging in the medial Golgi with limited α-mannosidase II activity to trim the mannoses on the α1,6-arm of the glycoconjugate, or upon addition of a bisecting GlcNAc before trimming takes place, as the presence of bisection inhibits the activity of α-mannosidase II (23). Hybrid-type glycans have been reported to be present on human polyclonal IgG (24, 25), albeit in minor amounts, and little is known about their specific effects on IgG-Fc function.

Similar to galactosylation, IgG1 sialylation was low in patients with hematological malignancies pre-HSCT as compared to both the healthy controls and donors. However, after HSCT, sialylation showed an increase, which was more pronounced when studied relative to the level of galactosylation. While pre-HSCT sialylation was lower than in healthy controls and in donors, post-HSCT no difference was observed between case and control or donor levels. In addition, post-HSCT sialylation per galactose was higher than the levels observed in the donors. While overall sialylation effects might be a possible side-effect of changing galactosylation (terminal galactoses are a substrate for sialylation), an increase in the amount of sialic acids per galactose is likely to be caused by the upregulation of the sialyltransferase ST6Gal1 or an increased availability of the substrate CMP-sialic acid (12). Next to the regulation inside the B cell by genetic and local factors (13, 15, 16), IgG Fc sialylation in mice is also reported to be dynamically regulated independently of the B cell (26). This is suggested to happen in a stress-driven way, with the accumulation of platelets that serve as source of CMP-sialic acid (27). In murine models, an increase in Fc sialylation was reported to act as an anti-inflammatory regulator resulting in upregulation of the FcγRIIb on macrophages (28). However, this finding is hard to translate directly to the human situation, due to the differences between mice and men in IgG effector functions (29). In human *in vitro* models, an increased Fc sialylation on polyclonal IgG was connected to a decreased C1q binding and, consequently, to an impairment of CDC (4).

While various association studies in humans point toward a more pronounced role of galactosylation rather than of sialylation in potentially mediating pro- and anti-inflammatory effects of IgG (30–32), these studies were all performed in the adult population, while knowledge about the effects in children is limited. A recent study showed the decrease of sialylation per galactose, but not of galactosylation, with age in healthy children (6). Contrary, for healthy adults it was reported that galactosylation decreases with age, while sialylation per galactose remains stable in males and only slightly decreases in females (5). These differences in the regulation of IgG galactosylation and sialylation between children and adults warrant further investigation as to the causes as well as downstream effects.

Both IgG1 hybrid-type glycans and sialylation showed a difference between the healthy controls or donors and the recipient treated for hematological malignancies pre-HSCT which was not observed anymore post-HSCT. This might indicate that environmental factors regulating these Fc glycosylation traits have been normalized.

### Disease-Specific IgG Fc Glycosylation

While for patients treated for hematological malignancies, pre-HSCT, multiple differences in IgG Fc glycosylation were observed as compared to healthy controls, for the group with non-malignant diseases, less profound differences were found between cases and controls. Specifically, lower levels of galactosylation and sialylation, as well as lower hybrid-type glycans, were exclusively observed in the group of hematological malignancies. It has previously been reported that IgG galactosylation decreased with different forms of cancer in adults, like ovarian and colorectal cancer (9, 10), while corresponding knowledge on malignancies in children has hitherto been lacking. To our knowledge, the only IgG glycosylation studies performed in diseased children so far were in patients with allergic diseases, asthma, and juvenile idiopathic arthritis, showing no associations between aberrant IgG glycosylation profiles and the two former disorders (33, 34), and a decreased galactosylation and sialylation in juvenile idiopathic arthritis (35). Here, we show that pediatric patients treated for hematological malignancies have an altered IgG Fc glycosylation, which might be specific for their condition or treatment regimens as compared to patients treated for non-malignant hematological diseases.

### Conclusion

While the B cells in children after HSCT are mainly of donor origin (36) and patients were investigated after reaching independency of IgG supplementation, we found that IgG Fc glycosylation in our pediatric cohort did not reflect the glycosylation pattern of the donors. Galactosylation was already low pre-HSCT in patients treated for hematological malignancies as compared to their donors and age-matched healthy controls, and remained at this lower level the first year after HSCT. In patients treated for non-malignant hematological diseases, galactosylation showed a strong decrease to similarly low levels after HSCT. These data suggest that external local influences on the IgG Fc glycosylation are operative before HSCT in patients treated for hematological malignancies as well long-term after HSCT independently of the original disease. To further identify the external factors influencing IgG glycosylation after HSCT, future research should focus on the expression of glycan-modifying enzymes in the ER and Golgi apparatus, and in addition on the molecules that can influence up- and down-regulation of these enzymes, like cytokines and hormones (15, 16). Furthermore, antigen-specific IgG glycosylation analysis, for example, of anti-pneumococcal or anti-meningococcal antibodies after vaccination or of autoantibodies, might shed light on the influence of antigen stimulation on the IgG glycosylation profile.

Other IgG1 glycosylation features like hybrid-type glycans and sialylation were also low prior to HSCT in the group treated for hematological malignancies, but normalized after transplantation. This could either be explained by normalization of environmental factors influencing the latter IgG Fc glycosylation traits upon curing the disease or by a reflection of the donor glycosylation status based on donor genetics. Future studies, in populations that contain both cases with and without post-HSCT complications like immunodeficiencies or antibody mediated autoimmunity, might reveal pathology-specific glycosylation profiles.

## Data Availability Statement

The raw data supporting the conclusions of this manuscript will be made available by the authors, without undue reservation, to any qualified researcher.

## Ethics Statement

This study was carried out in accordance with the recommendations of Medical Ethics Committee of the LUMC, Leiden and Medical Ethics Committee of the Erasmus MC, Rotterdam with written informed consent from all subjects. All subjects gave written informed consent in accordance with the Declaration of Helsinki. The protocol was approved by the Medical Ethics Committee of the LUMC, Leiden and Medical Ethics Committee of the Erasmus MC, Rotterdam. Corresponding project numbers were “project P01.028” for the clinical samples obtained with approval of the Medical Ethics Committee of the LUMC, Leiden and “MEC-2005-137” for the healthy control samples obtained with approval of the Medical Ethics Committee of the Erasmus MC, Rotterdam.

## Author Contributions

NH, MT, AL, and MW designed the study. NH performed sample preparation and experimental analysis. NH processed data, which were further analyzed by NH, MT, AL, and MW. GD collected the samples and clinical data of the healthy controls. NH, MT, AL, and MW drafted the manuscript and all authors read the final manuscript critically.

## Conflict of Interest Statement

The authors declare that the research was conducted in the absence of any commercial or financial relationships that could be construed as a potential conflict of interest.
