# Supplementary material for: Immunoglobulin G Fragment Crystallizable Glycosylation After Hematopoietic Stem Cell Transplantation Is Dissimilar to Donor Profiles
Source: Front Immunol. 2018 Jun 4;9:1238. doi: 10.3389/fimmu.2018.01238 (PMC5994695; doi:10.3389/fimmu.2018.01238)
Supplement: Supplementary file 1 [file Presentation_1.PDF]

Supplementary Material

IgG Fc Glycosylation After Hematopoietic Stem Cell Transplantation is Dissimilar to Donor Profiles

Noortje de Haan[1], Maarten J.D. van Tol[2], Gertjan J. Driessen[3,4], Manfred Wuhrer[1], Arjan C. Lankester[2,*]

[1] Center for Proteomics and Metabolomics, Leiden University Medical Center, Leiden, The Netherlands

[2] Department of Pediatrics, section Immunology, Hematology and Stem Cell Transplantation, Leiden University Medical Center, Leiden, The Netherlands

[3] Department of Pediatrics, Juliana Children's Hospital, Haga Teaching Hospital, The Hague, The Netherlands

[4] Department of Pediatrics, Erasmus Medical Center, Sophia Children's Hospital, Rotterdam, The Netherlands

*** Correspondence:**
Arjan C. Lankester
a.lankester@lumc.nl

Content

Figure S1. Repeatability of the method.

Figure S2. The use of plasma and serum samples results in comparable IgG Fc-glycosylation profiles.

Figure S3. IgG Fc-Glycosylation features in all patients, compared to their donors and age-matched healthy controls.

Figure S4. IgG Fc-Glycosylation features in patients transplanted for hematological malignancies, compared to their donors and age-matched healthy controls.

Figure S5. IgG Fc-Glycosylation features in patients transplanted for non-malignant hematological diseases, compared to their donors and age-matched healthy controls.

Supplementary Materials and Methods

Supplementary Tables S1 to S5 (separate Excel file)

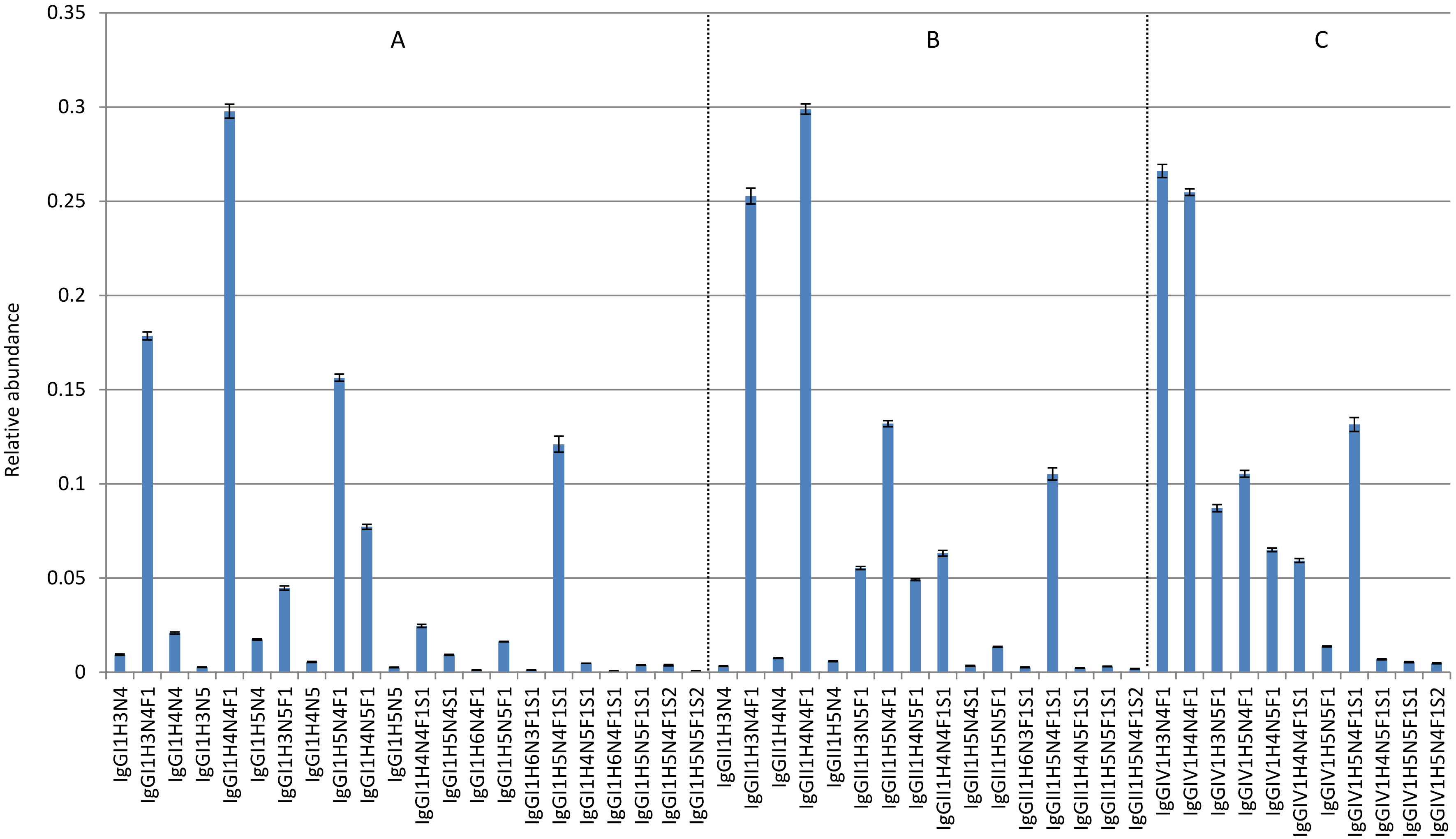

Figure S1. Repeatability of the method. Relative abundances of the extracted IgG1 (**A**), IgG2/3 (**B**) and IgG4 (**C**) glycoforms in the 29 pooled plasma standards included in the healthy control cohort and the 12 pooled plasma standards included in the clinical sample cohort. Shown are the average relative abundances and standard deviations over all technical replicates, revealing highly repeatable profiles throughout both cohort measurements. H: Hexose, N: *N*-acetylhexosamine, F: Fucose, S: *N*-acetylneuraminic acid, IgGI: IgG1 glycopeptide, IgGII: IgG2/3 glycopeptide, IgGIV: IgG4 glycopeptide.

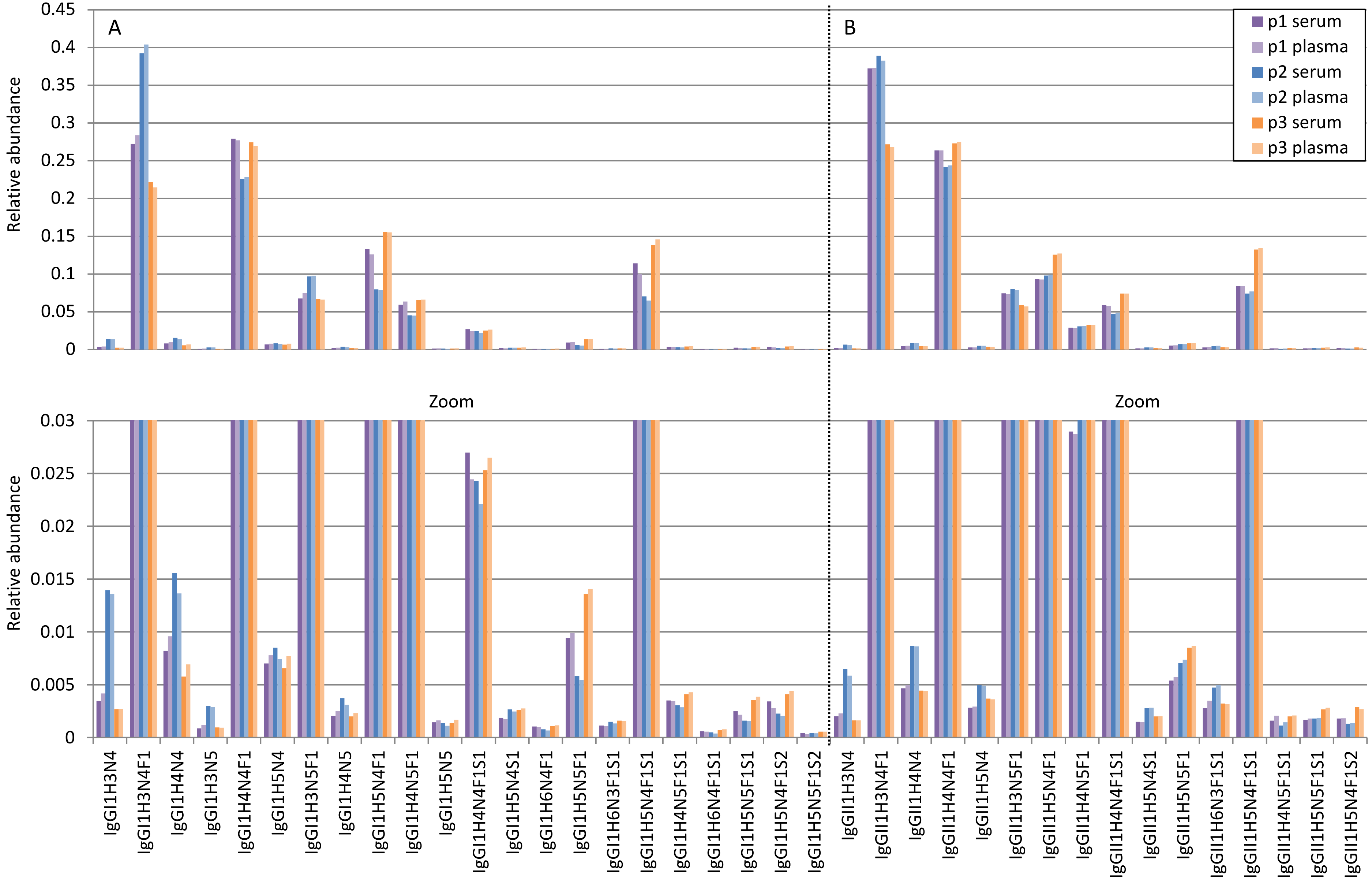

Figure S2. The use of plasma and serum samples results in comparable IgG Fc glycosylation profiles. Relative abundances of the extracted IgG1 (**A**) and IgG2/3 (**B**) glycoforms in the three patients pre-HSCT for which both plasma and serum samples were available. Serum samples (darker color) showed comparable IgG Fc glycosylation profiles to plasma samples (lighter color). The difference between plasma and serum from the same patient was lower than the variation between patients, enabling the use of both materials in this study. H: Hexose, N: *N*-acetylhexosamine, F: Fucose, S: *N*-acetylneuraminic acid, IgGI: IgG1 glycopeptide, IgGII: IgG2/3 glycopeptide, IgGIV: IgG4 glycopeptide.

Figure S3. IgG Fc Glycosylation features in all patients, compared to their donors and age-matched healthy controls. H; healthy controls, Pre: patients prior to HSCT, 6 m: patients six months after HSCT, 12 m: patients twelve months after HSCT, D: donors prior to graft donation.

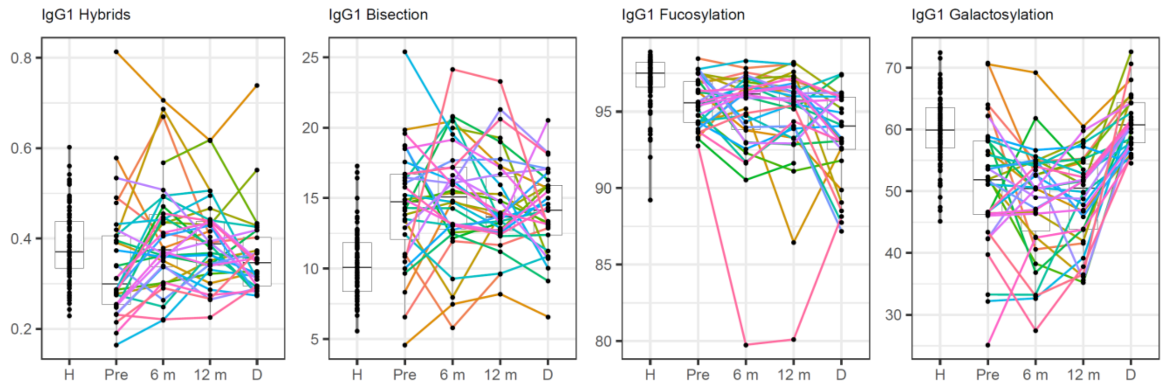

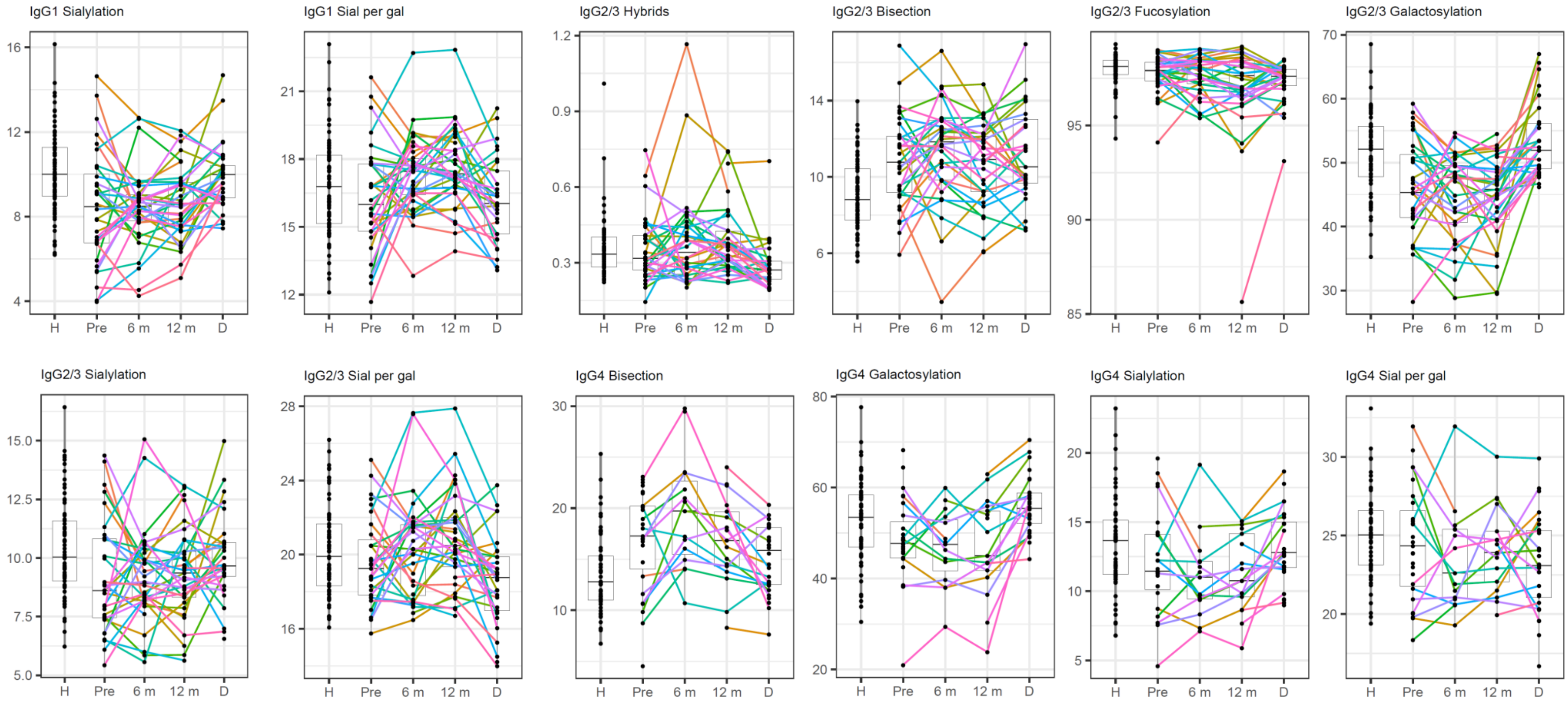

Figure S4. IgG Fc Glycosylation features in patients transplanted for hematological malignancies, compared to their donors and age-matched healthy controls. H; healthy controls, Pre: patients prior to HSCT, 6 m: patients six months after HSCT, 12 m: patients twelve months after HSCT, D: donors prior to graft donation.

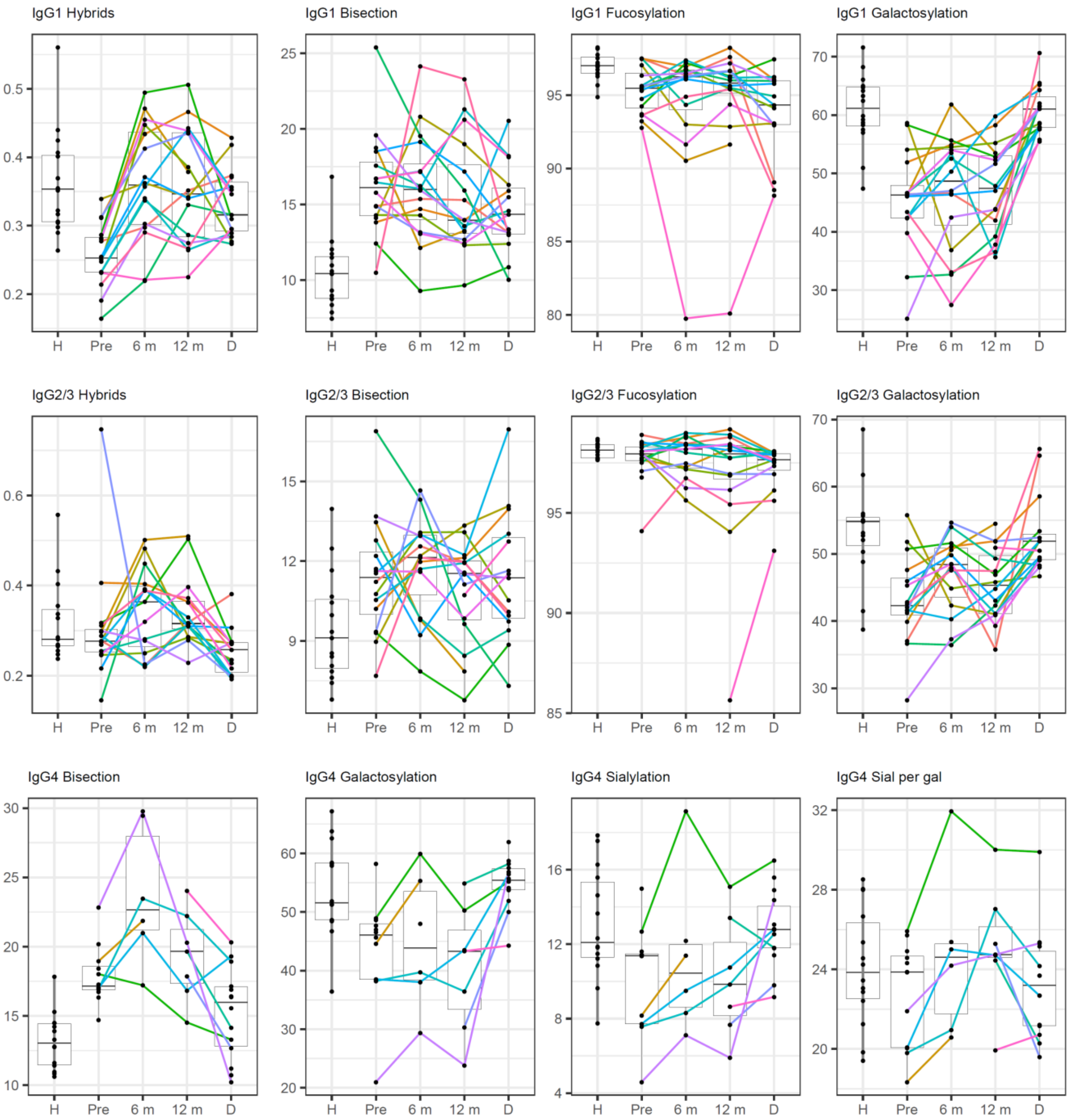

Figure S5. IgG Fc Glycosylation features in patients transplanted for non-malignant hematological diseases, compared to their donors and age-matched healthy controls. H; healthy controls, Pre: patients prior to HSCT, 6 m: patients six months after HSCT, 12 m: patients twelve months after HSCT, D: donors prior to graft donation.

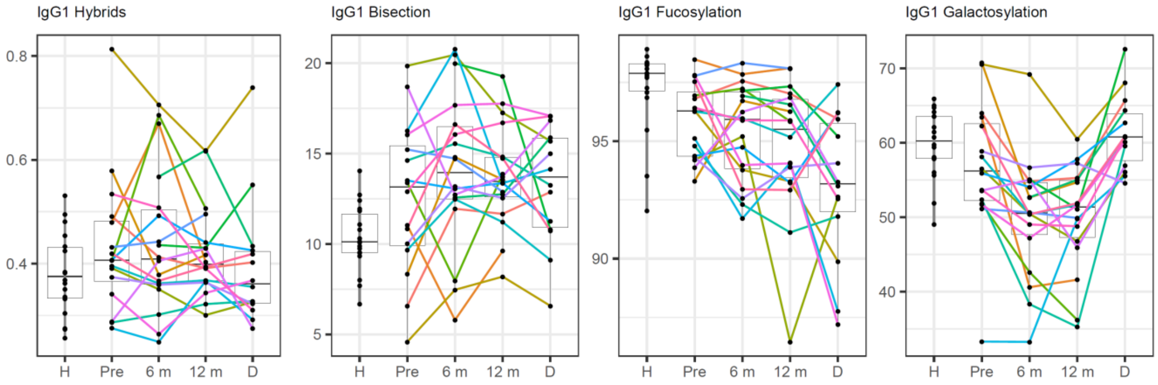

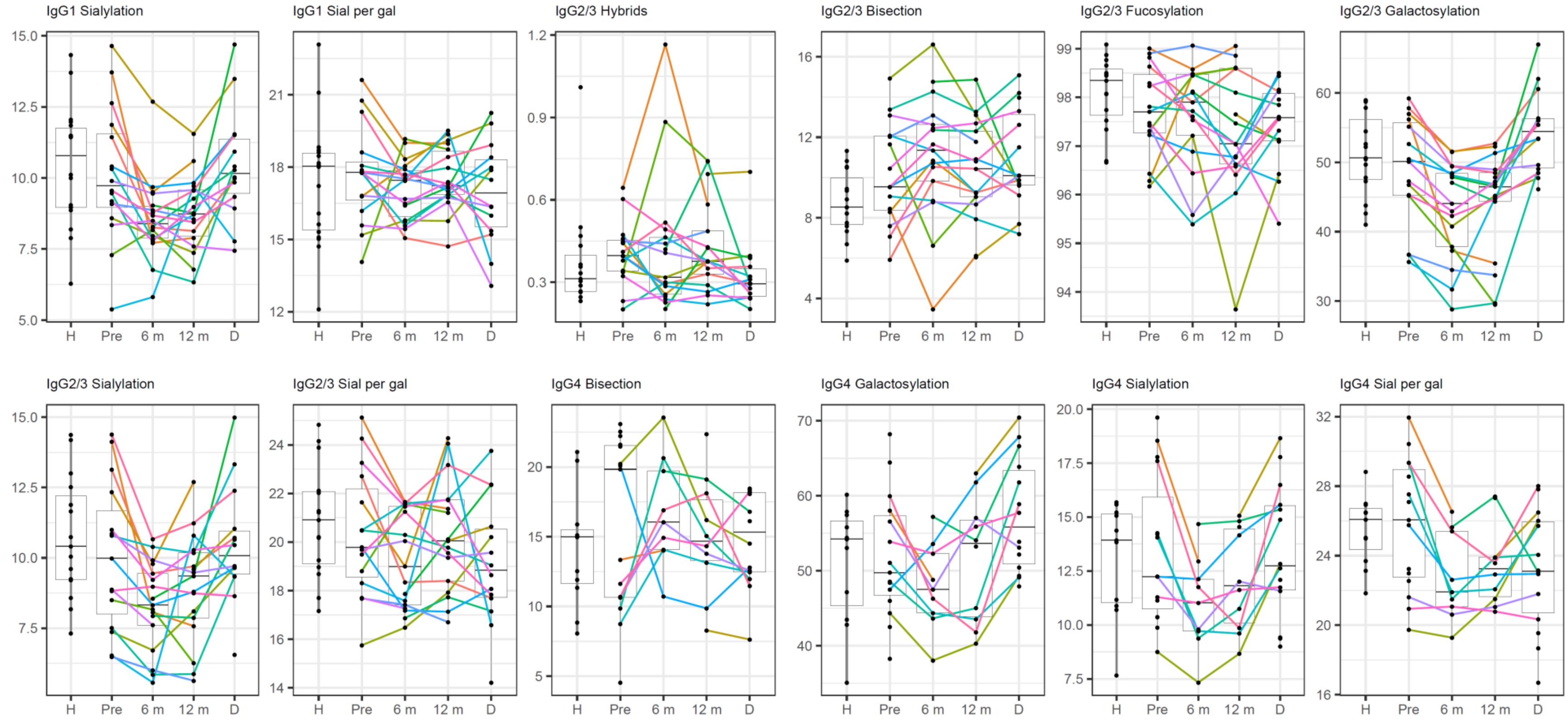

Supplementary Materials and Methods

Chemicals

Ultra-pure deionized water (MQ) was generated by the Purelab Ultra, maintained at 18.2 MΩ (Veolia Water Technologies Netherlands B.V., Ede, The Netherlands) and used throughout the study. Disodium hydrogen phosphate dihydrate ($Na_2HPO_4 \cdot 2H_2O$), potassium dihydrogen phosphate (KH_2PO_4), NaCl, and trifluoroacetic acid were purchased from Merck (Darmstadt, Germany). Formic acid, ammonium bicarbonate, and TPCK-treated trypsin from bovine pancreas were obtained from Sigma-Aldrich (Steinheim, Germany). Furthermore, HPLC SupraGradient acetonitrile (ACN) was obtained from Biosolve (Valkenswaard, The Netherlands) and phosphate-buffered saline (PBS) was made in-house, containing 5.7 g/L $Na_2HPO_4 \cdot 2H_2O$, 0.5 g/L KH_2PO_4 and 8.5 g/L NaCl.

IgG Isolation and Glycopeptide Preparation and Analysis

All 135 clinical samples (patients and donors) were randomized on two 96-well plates, together with 12 VisuCon pooled plasma standards (Affinity Biologicals Inc., Ancaster, ON, Canada) and 5 PBS blanks. Healthy control samples were randomized on separate plates, including 29 VisuCon pooled plasma standards and 8 PBS blanks. IgG was isolated using Protein G affinity beads (GE Healthcare, Uppsala, Sweden) as described before (1). Briefly, 2 µL of plasma was incubated with 15 µL of beads in 100 µL of PBS for 1 h with agitation. Samples were then washed three times with 200 µL of PBS and three times with 200 µL of MQ, after which the antibodies were eluted from the beads with 100 µL 100 mM formic acid. The eluates were dried for 2 h at 60 ˚C in a vacuum concentrator and redissolved in 40 µL 25 mM ABC containing 1 µg trypsin. Samples were shaken for 10 min and incubated at 37 ˚C for 17 h. The IgG digest was separated and analyzed by nano-liquid chromatography (LC) coupled by electrospray ionization to a Maxis Impact HD quadrupole time-of-flight mass spectrometer (q-TOF-MS; Bruker Daltonics, Bremen, Germany) as described before (1). The HPLC system consisted of a gradient pump, a isocratic loading pump, an autosampler maintained at 4 °C and a column oven maintained at 30 °C. Of each sample 250 nL was injected into a flow of 25 µL/min of solvent A (aqueous 0.1 % TFA (v/v)) and trapped on the trap column (Dionex Acclaim PepMap100 C18, 5 mm × 300 µm; Thermo Fisher Scientific, Breda, The Netherlands). With a flowrate of 900 nL/min the analytes were eluted on a C18 nano-column (Ascentis Express C18 nanoLC column, 50 mm × 75 µm, 2.7 µm fused core particles; Supelco, Bellefonte, PA) and separated in a linear gradient from 3% to 30% solvent B (95% ACN (v/v)) in 5 min. The interface between the HPLC system and the q-TOF-MS was achieved with a nanoBooster (Bruker Daltonics), using ACN-doped nebulizing gas (pressure: 0.2 bar; dry gas flow: 3.0 L/min; dry temperature: 180 °C). Profile spectra were recorded in *m/z* range 550 to 1800 with a frequency of 1 Hz. The collision energy was 7.0 eV, the transfer time 110 µs, and the pre-pulse storage 21 µs. The total analysis time per sample was 12 min. The HPLC system and the q-TOF-MS were operated under Chromeleon Client v6.80 build 3161 and otofControl v3.4 build 14, respectively.

Data Processing

The raw LC-MS data were extracted and curated using the in-house developed software LacyTools v0.0.7.2 as described previously (1, 2). Briefly, the chromatograms of each run were aligned based on the exact mass and the average retention time over all runs of the three

most abundant glycoforms of each IgG subclass; H3N4F1, H4N4F1 and H5N4F1. An alignment time window of ±10 s and a mass window of ±0.1 Th were used. Glycopeptide peaks were used for alignment only when their signal-to-noise ratio (S/N) was above nine, with a minimum of seven glycopeptides per sample. Using the described separation methods, glycopeptides with the same peptide portion co-eluted for most samples. This resulted in three glycopeptide clusters: one for IgG1, one for IgG4 and one for the combination of IgG2 and 3. The tryptic Fc glycopeptides for IgG2 and 3 have identical peptide moieties in the Caucasian population and are therefore not distinguishable by this profiling method. After alignment, sum spectra were created per glycopeptide cluster, with a time window of ±12 s per cluster. Sum spectra were calibrated based on at least four glycopeptides per cluster with a S/N higher than nine; for all IgG subclasses H3N4F1, H4N4F1, H5N4F1, H5N5F1 and H5N4F1S1 were used for calibration. The mass window used for calibration was 0.3 Da. For the targeted extraction, analyte lists were created by manual annotation of summed spectra per biological class (healthy, pre-transplantation, 12 months post-transplantation or donor), covering both doubly charged and triply charged species. Compositional assignments were made based on accurate mass in MS and literature (3-6). Glycopeptide signals were integrated by including enough isotopomers to cover at least 99% of the area of the isotopic envelope. Background subtraction was performed based on local background calculations. Spectra were excluded from further analysis when the total spectrum intensity was below ten times the average spectrum intensity of the blanks. In this way, no spectra were excluded for IgG1, 16 spectra were excluded for IgG2/3 and 64 spectra were excluded for IgG4. Analytes were included in the final data analysis when their average S/N (calculated per biological class) was above nine, their isotopic pattern did not, on average, deviate more than 20% from the theoretical pattern and their average mass error was between -10 and 10 ppm. This resulted in the extraction of 22 IgG1, 16 IgG2/3 and 11 IgG4 glycoforms (**Table S1** in Supplementary information).

1. Falck D, Jansen BC, de Haan N, Wuhrer M. High-Throughput Analysis of IgG Fc Glycopeptides by LC-MS. *Methods in molecular biology* (2017) **1503**:31-47. doi: 10.1007/978-1-4939-6493-2_4. PubMed PMID: 27743357.
2. Jansen BC, Falck D, de Haan N, Hipgrave Ederveen AL, Razdorov G, Lauc G, et al. LaCyTools: A Targeted Liquid Chromatography-Mass Spectrometry Data Processing Package for Relative Quantitation of Glycopeptides. *Journal of proteome research* (2016) **15**(7):2198-210. doi: 10.1021/acs.jproteome.6b00171. PubMed PMID: 27267458.
3. Bondt A, Rombouts Y, Selman MH, Hensbergen PJ, Reiding KR, Hazes JM, et al. Immunoglobulin G (IgG) Fab glycosylation analysis using a new mass spectrometric high-throughput profiling method reveals pregnancy-associated changes. *Molecular & cellular proteomics : MCP* (2014) **13**(11):3029-39. doi: 10.1074/mcp.M114.039537. PubMed PMID: 25004930; PubMed Central PMCID: PMC4223489.
4. de Haan N, Reiding KR, Driessen G, van der Burg M, Wuhrer M. Changes in Healthy Human IgG Fc-Glycosylation after Birth and during Early Childhood. *Journal of proteome research* (2016) **15**(6):1853-61. doi: 10.1021/acs.jproteome.6b00038. PubMed PMID: 27161864.
5. Pucic M, Knezevic A, Vidic J, Adamczyk B, Novokmet M, Polasek O, et al. High throughput isolation and glycosylation analysis of IgG-variability and heritability of the IgG glycome in three isolated human populations. *Molecular & cellular proteomics : MCP* (2011) **10**(10):M111 010090. doi: 10.1074/mcp.M111.010090. PubMed PMID: 21653738; PubMed Central PMCID: PMC3205872.
6. Stumpo KA, Reinhold VN. The N-glycome of human plasma. *Journal of proteome research* (2010) **9**(9):4823-30. doi: 10.1021/pr100528k. PubMed PMID: 20690605; PubMed Central PMCID: PMC2933516.
